# The Rational Treatment of Pyrexia in Phthisis

**Published:** 1906-04-28

**Authors:** H. Hyslop Thomson


### Hospital Clinics.

### THE RATIONAL TREATMENT OF PYREXIA IN PHTHISIS.

By H. Hyslop Thomson, M.D.

The presence of fever in phthisis usually indicates progressive activity in the morbid processes in the lungs. Occasionally in acute manifestations of the disease fever is absent, while in the early phases of its development the temperature is frequently subnormal. Generally speaking, however, the extent and type of the fever bear a distinct relationship to the activity of the morbid process, and the amount of systemic intoxication which is being produced thereby. All types of fever may be met with in tuberculosis of the lungs. In acute pneumonia phthisis, general miliary tuberculosis, and widespread and rapidly progressing tubercule of the lung continued fever is the type usually observed. Marked hectic or intermittent fever points to the presence of cavities and a severe systemic intoxication, while fever of the remittent type is frequently seen in cases in which the tuberculous process is advancing and rapidly breaking down.

Apart from the pyrexia of nervous origin, or that due to the presence of complications or associated disease, the fever in most cases can be referred to the absorption into the systemic circulation of the toxins resulting from bacterial growth. The bacteria are the tubercle bacilli with which are frequently associated staphylococci, streptococci, and other pyogenic micro-organisms. Occasionally the condition is more severe than that of toxæmia, for the entrance of micro-organisms into the bloodstream will cause, in the case of pyogenic bacteria, septicæmia, and in the case of the bacilli of tubercle acute miliary tuberculosis.

The importance of directing efforts towards the reduction of fever in cases of acute or sub-acute phthisis becomes obvious when we bear in mind that pyrexia impairs the functions of digestion and assimilation, and is instrumental in lowering the resistance of the tissues to the development of the tuberculous processes. By this means we have established a vicious circle.

The treatment of fever in phthisis should consist of a fourfold effort: (1) to secure respiratory rest; (2) to improve the nutrition and raise the resistance of the tissues; (3) to diminish bacterial activity; and (4) to directly influence the fever by antipyretic remedies.

### Rest.

In the treatment of the pyrexia in phthisis, the first and most important measure to adopt is rest in bed. The importance of rest in dealing with the fever associated with active pulmonary tubercle will be readily appreciated when we call to mind the good results obtained by fixation in the treatment of tuberculous joints. Although, unfortunately, we cannot obtain absolute pulmonary rest, yet we can procure it in relative degree by keeping the patient confined to bed in a single room and suppressing all exciting and disturbing factors. Muscular activity, boisterous mirth, incessant talking, and loud laughter are contra-indicated, as they are followed by increased respiratory movement, and consequently may give rise to further auto-infection.

In prescribing rest in bed and isolation either in a room or shelter, it is advisable not to adopt any hard or fast rule, but to consider each individual case on its merits. Speaking generally, when the temperature taken in the mouth or rectum exceeds 99° F. in the morning, or 100.4° F. in the afternoon or evening, rest and perfect quietness should be enjoined. In slight grades of pyrexia this alone frequently suffices in due course to reduce the temperature to within normal limits. One important point is that nervous patients should be kept in total ignorance of the variations in their temperature, and on no consideration be allowed to study the chart.

Occasionally, in patients who unfortunately are in possession of a nervous and anxious temperament, isolation in bed appears to give rise to much mental distress, more especially when the feeling of well-being is not much reduced below normal, and in consequence of this physical rest fails to have the desired effect. In such cases it is well to break through the customary rule and allow the patients to get up and rest in some secluded spot out of doors, either on a couch or camp chair.

The importance of avoiding any rigid adherence to rule-of-thumb methods in the treatment of fever in phthisis is well illustrated by the following case:

A young man who had been resident in a sanatorium for some months, and was regarded as apparently cured, had a somewhat sharp hæmoptysis a few weeks after leaving. He was thoroughly conversant with the details of treatment, which he continued to carry out at home in a very efficient manner. Following the hæmorrhage there was some degree of fever, associated with cough, expectoration, and dyspeptic symptoms. He was ordered to remain in bed, and at my request registered his temperature on a chart morning, afternoon, and evening. At the end of a month his condition showed no signs of improvement, but was rather worse. The mean temperature was a degree higher, he looked thinner, and whereas before the onset of hæmorrhage he had been fit and well, he was now feeling ill and shaky, and had become very despondent as to the prospects of recovery. His mother informed me that he was worrying a great deal about his temperature, and that it was not unusual for him to take it five or six times a day.

As the disease was limited to the left upper lobe, and the dyspeptic symptoms had yielded to treatment, I took the chart and thermometer from him, and allowed him to rise at 10 A.M. and rest in a large room by the open window until 4 P.M., and to do this for a week. During the second week I prescribed a short walk every afternoon; this was gradually increased during the third week, according as his strength and condition generally permitted. From the date of rising he has steadily improved, has increased several pounds in weight, and the temperature on the occasions when it has been taken has been very rarely above 100° F. in the evening. In a word, the result has amply justified the unusual departure from the recognised line of treatment.

### Diet.

In the treatment of fever much depends upon the condition of the gastro-intestinal tract and the capacity for digestion. If the normal processes of digestion and absorption are interfered with for any length of time, the nutrition of the tissues suffers and their resisting powers are enfeebled, so that the morbid processes become more active and the toxic output is correspondingly increased. Conversely, improved nutrition and increased resistance will tend to retard the development of the pulmonary lesion and restrict the amount of auto-infection. It is mainly for this reason that the question of diet occupies an important place in the treatment of pulmonary tuberculosis.

The dieting of patients with well-marked pyrexia calls for care and discrimination. When the temperature does not greatly exceed 101° F. at any time of the day the patient may be given the full sanatorium diet. This, of course, varies in different institutions both as to quantity and variety; but experimentally it has been proved by Chapman and Bardswell [1] that a daily diet of between 2,000 and 3,000 caloric value will amply suffice. The heat equivalent of the full diet in use at the sanatorium at Bridge of Weir is a little over 3,000 calories.

The effect of dietetic treatment in these cases must be carefully watched. Immediately symptoms of deranged digestion appear everything must be subservient to the effort to re-establish the normal digestive function. To persist in giving a full diet under such circumstances will not only defeat the end in view, but will probably aggravate the condition, by promoting fermentative changes in the intestines, and so lead to heightened fever from auto-intoxication. Parenthetically it may be here remarked that septic changes in the intestinal tube may in themselves be the direct cause of frequent pyrexia excursions in phthisis. Fever due to this cause usually comes on suddenly, in a patient who has been for some time under open-air treatment, with such symptoms as nausea, vomiting, abdominal discomfort or actual pain, and frequent offensive stools. Such symptoms, associated with a sudden rise in temperature, call for a modification of diet and the administration of a purge, such as calomel, followed by a saline, after which some drug possessing antiseptic properties, such as salol, bismuth salicylate, or benzoate of soda should be given.

When the temperature as a result of toxic influences rises regularly above 102° F. at some hour of the day, the diet to begin with should be of a light and easily assimilable nature. The best diet in these cases consists of light soups, milk, raw beef juice, fish, rabbit, chicken, tripe, and milk pudding. In the higher phases of fever with meagre digestive capacity even this diet may not be well borne, in which case liquid nourishment alone should be given, consisting for the most part of light soups, raw beef juice, and milk. Or as an alternative the patient may be restricted to a raw diet, made up of raw eggs, minced raw beef, beef juice, and oysters. Certainly, in some cases of phthisis with well-marked pyrexia, raw beef and raw beef juice are better tolerated than cooked food.

### Antiseptic Treatment.

This includes the adoption of certain measures to limit bacterial activity, so as to secure some restriction of the toxic output, the extent of which stands in such close relationship to the amount of fever. In cases of mixed infection the activity of the pus organisms is no doubt lessened to some extent by the continued respiration of pure sterile air, but their destruction can only be attained with the aid of certain antiseptic drugs, exhibited by the mouth, by inhalation, by inunction, or by the combined method. Of drugs given by the mouth for this purpose creosote has proved by far the most useful. It should be given in gradually increasing doses, from two to five minims upwards, thrice daily, after meals. It may be taken dissolved in spt. vini rect. and spt. chloroformi, or emulsified in mucilage acaciæ and glycerin. When not well tolerated it may be replaced by thiocol, guaiacol, or guaiacol carbonate; or it can be administered advantageously by inunction in combination with essential oils, as follows: ℞ creosoti ʒij., ol. pini ℥ss., ol. eucalypti ℥ss., ol. caryophylli ℥ss., ol. olivæ ℥ss., ol. camph. ℥ss. Sig.: ʒj. to ʒij.; to be well rubbed in night and morning. Along with the administration of creosote and the inunction of essential oils it is well to combine some form of antiseptic inhalation, especially if cough is severe and there is much purulent expectoration. Inhalations of formalin do good in some cases. The following combination will be found serviceable: ℞ menthol ʒj., creosoti ʒj., chloroform ℥j. Sig.: ♏x. to ♏xx.; to be inhaled several times daily.

By the combined method of administering creosoti or guaiacol with the inunction of essential oils and the inhalation of volatile antiseptics we will be able to inhibit to some extent, if not actually destroy, the activity of the pus organisms, which are mainly responsible for high grades of fever.

Another method worthy of trial is the local exhibition of antiseptics by intra-tracheal injections. In several cases of mixed infection in which these injections were given some definite amelioration of the degree of fever present was observed. A solution of menthol, guaiacol, and iodoform in olive oil is most commonly used, but Colin Campbell, who has largely employed this line of treatment, recommends the use of medicated izal in glycerin. The same authority points out that immediately after the injection has been given the patient should be made to lie down on the affected side, when the condition is unilateral, so as to encourage the flow of the antiseptic into the bronchus of the diseased lung.

The advance which has been made within recent years in the region of serum-therapy raises the question of the possible treatment of toxic manifestations in phthisis by a suitable serum. In cases complicated by streptococcic infection the antistreptococcic serum should be given a trial, although opinions differ widely as to the influence which it exercises on the temperature. Marmoreck strongly recommends its use in combination with his anti-tuberculous serum in cases characterised by hectic fever.

Anti-diphtheric serum administered by the mouth or subcutaneously will occasionally be found to lower the temperature, to some extent, in cases of mixed infection. I have in mind one case especially, in which this serum was the only agent which influenced the fever.

Occasionally in phthisis the presence of fever is in part due to some condition with which it is possible to deal directly. Thus it may depend primarily upon a septic condition of the mouth, the treatment of which by an antiseptic mouth-wash and the removal of decayed teeth will usually be followed by an appreciable decrease in the amount of pyrexia. Pyorrhœa alveolaris is sometimes met with as a complication in phthisis. During the acme of the suppurative condition in the mouth the fever is usually high, but it can be appreciably lowered by energetic antiseptic treatment of the local condition. It must be admitted, however, that the ultimate result in cases of phthisis complicated by Rigg's disease will turn out to be disappointing.

Antipyretic Measures.

When the temperature in phthisis continues elevated and has failed to be influenced by rest and isolation it may be necessary, in order to break through the vicious circle, to induce a definite fall in temperature by employing direct antipyretic measures. Before resorting to drugs, cold sponging and the application of cold compresses should be given a somewhat extended trial. For this purpose the temperature must be taken every three hours, and when a rise of 102° F. or more is recorded, the surface of the body should be sponged with ice-water and a cold compress applied over the thorax and abdomen. At first some objection may be raised by the patient to this line of treatment, but he will very soon come to appreciate its grateful effect. Neimeyer's pill, containing digitalis, quinine, and opium may be usefully combined with the cold applications, one being taken three times a day.

Of the antipyretic drugs which may be given, the most likely to produce a definite result are quinine, antipyrin, aspirin, pyramidon, and cryogenine. The salts of quinine, the sulphate, the bi-hydrochloride, or the salicylate sometimes answer well in cases in which the temperature rises suddenly with a feeling of chill or a slight rigor. Aspirin and pyramidon, however, have in my experience yielded the best results. If the early morning temperature is not much elevated, but there is a well-marked rise towards the afternoon or evening, I usually prescribe one or other drug in doses varying from 5 to 15 grains twice daily, about 11 a.m. and 3.30 p.m. Occasionally pyramidon produces a feeling of nausea and some slight depression, but this effect is rarely so marked as to contra-indicate its use. I have seen some very striking results following the exhibition of this drug. Cryogenine is very highly spoken of by some Continental authorities as an antipyretic in the fever of phthisis. It is stated to be efficacious in small doses, and to give rise to no ill effects, even if taken for a lengthened period.

One important point to remember in the treatment of pyrexia in phthisis is that antipyretic remedies are not likely to be followed by any definite beneficial result if used alone; they must, when employed, especially in cases of mixed infection, be combined with some form of antiseptic treatment which has as its aim the destruction of pyogenic bacteria.

There is no doubt a wide field for observation and research work in the mixed infections met with in the course of pulmonary phthisis. The behaviour of the tubercle bacilli in the presence of pyogenic bacteria is but little known. In some advanced cases the expectoration contains large numbers of pyo-cocci, while tubercle bacilli are present in small numbers or absent altogether. One point, however, seems well established, that the exclusion of pus organisms from the diseased area would render the treatment of the pulmonary lesion a much less tedious and more satisfactory problem than it is at present.

[1] Brit. Med. Jour., Feb. 22, 1902.

